# Targeting Reactive Oxygen Species Capacity of Tumor Cells with Repurposed Drug as an Anticancer Therapy

**DOI:** 10.1155/2021/8532940

**Published:** 2021-09-07

**Authors:** Jiabing Wang, Dongsheng Sun, Lili Huang, Shijian Wang, Yong Jin

**Affiliations:** ^1^Municipal Hospital Affiliated to Taizhou University, Taizhou 318000, China; ^2^Taizhou University Hospital, Taizhou University, Taizhou, Zhejiang 318000, China; ^3^Lihuili Hospital Affiliated to Ningbo University, Ningbo, Zhejiang 315100, China

## Abstract

Accumulating evidence shows that elevated levels of reactive oxygen species (ROS) are associated with cancer initiation, growth, and response to therapies. As concentrations increase, ROS influence cancer development in a paradoxical way, either triggering tumorigenesis and supporting the proliferation of cancer cells at moderate levels of ROS or causing cancer cell death at high levels of ROS. Thus, ROS can be considered an attractive target for therapy of cancer and two apparently contradictory but virtually complementary therapeutic strategies for the regulation of ROS to treat cancer. Despite tremendous resources being invested in prevention and treatment for cancer, cancer remains a leading cause of human deaths and brings a heavy burden to humans worldwide. Chemotherapy remains the key treatment for cancer therapy, but it produces harmful side effects. Meanwhile, the process of de novo development of new anticancer drugs generally needs increasing cost, long development cycle, and high risk of failure. The use of ROS-based repurposed drugs may be one of the promising ways to overcome current cancer treatment challenges. In this review, we briefly introduce the source and regulation of ROS and then focus on the status of repurposed drugs based on ROS regulation for cancer therapy and propose the challenges and direction of ROS-mediated cancer treatment.

## 1. Introduction

As a common and frequently occurring disease worldwide, cancers increasingly continue to produce serious clinical and socioeconomic issues [1, 2]. Reducing cancer mortality is the primary challenge globally, and the study on cancer treatment has increasingly been a hot spot in the field of scientific research [1, 2]. Despite the progress made in cancer therapy, the growing burden of the most common cancers in low-income and middle-income countries remains to be a major challenge [3]. More importantly, global cancer mortality is not much decreased compared with those in the past decades, though many new anticancer drugs have been approved for tumor prevention or treatment [4]. Unfortunately, the commonly used chemotherapeutics are accompanied with severe adverse effects [5]. Extensive efforts have been made to develop novel and highly efficacious tumor-targeting agents [6]. However, it is not only the frequent appearance of resistance concomitant with targeted therapies but also the higher budgets of targeted drugs that account for the limited use clinically, which lead to the classical cytotoxic drugs to remain the first choice for patients [7, 8]. Therefore, there is still a need to develop more effective and less toxic anticancer drugs worldwide to prevent and treat cancer.

**Figure 1:**
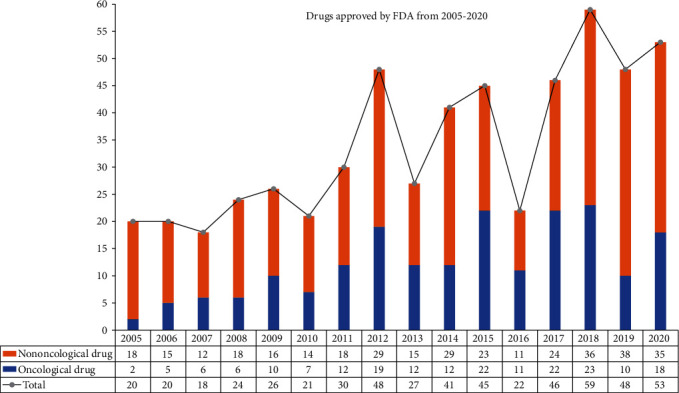
The number of the FDA approved drugs for oncology from 2005 to 2020.

Over the past few decades, the challenges of drug discovery facing the global pharmaceutical industry are multifold and stagnant, including the escalating cost and length of time required for new drug development and high risk of research and development failure [9, 10]. Currently, the cost of discovering and developing a drug from scratch is traditionally about 2.5 billion US dollars on average, and it takes about 10 to 15 years to enter the market and the success rate is only 2% [11, 12]. Few new anticancer drugs are approved by the FDA annually (Figure 1), though more than 10,000 clinical trials have been completed to evaluate cancer drug interventions [13]. Hence, alternative approaches to drug development are direly needed.

**Figure 2:**
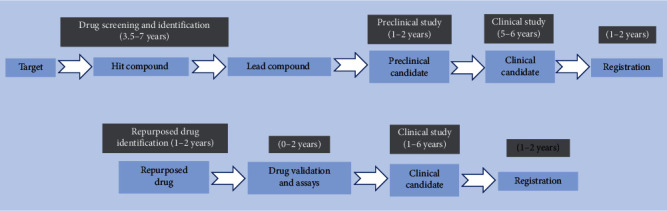
A comparison of the estimated time and main steps in de novo drug development and drug repurposing tactics.

In light of these challenges, drug repurposing may be an alternative approach to overcome obstructions and has gained much attention and momentum in recent years. Drug repurposing is the practice of discovering novel effects or targets of the approved drugs beyond their initial approval, which can expand the indications for marketed drugs [14]. Drug repurposing has many advantages over developing an entirely new drug for a specific indication. For example, repurposed drugs have been found to be safe enough in preclinical models and humans; it is unlikely to fail in early-stage trials and subsequent efficacy trials based on the safety standpoint [15]. Additionally, the time frame and investment for drug development can be reduced; that is, the return on investment in the development of repurposed drugs for new uses is more rapid (Figure 2) [15]. Finally, repurposed drugs may reveal new anticancer targets and pathways that can be further developed [15]. Historically, drug repurposing was largely discovered by accident by researchers [15]. Once it is discovered that repurposed drugs have off-target effects or newly discovered target effects, they will be developed commercially [15]. The most dramatic examples of repurposed drug are thalidomide and sildenafil citrate. Thalidomide was initially used to treat morning sickness, but it was found to cause severe skeletal birth defects in newborns [16]. However, it was successfully repositioned for use in erythema nodosum leprosum and multiple myeloma therapy [17]. Sildenafil citrate was originally developed as an antihypertension drug, but when Pfizer reintroduced it to treat erectile dysfunction and marketed it as Viagra [18]. Surprisingly, it captured vast majority of the erectile dysfunction drug market [15]. Such successes have encouraged the global pharmaceutical industry and drug researchers to identify repurposed drugs. Indeed, there is no systematic approach to predict which drugs can be used as repurposed drugs. The strategies towards identifying drug repurposing opportunities based on a number of promising candidate drugs roughly include computational and experimental approaches [15, 19]. A full account of the comprehensive strategies used for drug repurposing is beyond the scope of this review, and readers are directed elsewhere [15, 19].

## 2. Overview Biological Characteristics of Reactive Oxygen Species (ROS)

### 2.1. The Types and Sources of ROS

**Figure 3:**
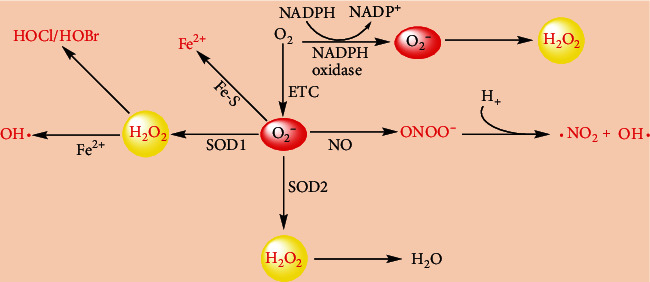
General scheme for ROS production by cellular enzymes and electron transport chain. The major sources of intracellular ROS include the mitochondrial ETC and NADPH oxidases. SOD1 and SOD2 can convert O_2_^−^ into H_2_O_2_; then H_2_O_2_ can be converted into H_2_O. Meanwhile, H_2_O_2_ can also be converted into OH^∙^, HOCl, and HOBr by Fe^2+^ and myeloperoxidase, respectively. NO is responsible for the conversion of O_2_^−^ into ONOO^−^ and OH^∙^.

In recent years, the understanding of tumor pathophysiology and pathogenesis has witnessed an unprecedented explosion. A large number of pleiotropic physiological signaling pathway factors regulating tumor occurrence and development have been emerged. Reactive oxygen species (ROS), the inevitable product of cell metabolism in aerobic life, are broadly defined as oxygen-containing chemical species with reactive properties, and they can be divided into nonradical and free radical [20, 21]. ROS are constantly produced by both enzymatic reaction and the mitochondrial electron transport chain (ETC) from molecular oxygen [20–22]. Complexes I, II, and III of mitochondrial ETC account for a great amount of the intracellular ROS production [23]. The enzyme-catalyzed reactions involve NADPH oxidase (NOX), xanthine oxidase, uncoupled endothelial nitric oxide synthase (eNOS), arachidonic acid, and metabolic enzymes such as the cytochrome P450 enzymes, lipoxygenase, and cyclooxygenase; indeed, NOX has primarily evolved to produce ROS [22, 24]. During the process of aerobic respiration and cellular metabolism, superoxide (O_2_^−^) is generated either intracellularly by 1 e^−^ transfer to O_2_ from the ETC or extracellularly by NOX. In the mitochondria, O_2_^−^ damages iron-sulfur (Fe-S) clusters to release iron (Fe^2+^) into the extracellular matrix and reduces ferric iron (Fe^3+^) to ferrous iron (Fe^2+^), which leads to inactivation of protein function [25, 26]. The O_2_^−^ is dismutated to hydrogen peroxide (H_2_O_2_) in a buffer or catalyzed by superoxide dismutases (SOD1 and SOD2) [22]. Moreover, H_2_O_2_ is also generated by various other oxidases present in subcellular localizations, prominently including the endoplasmic reticulum (ER) lumen [27, 28]. Meanwhile, O_2_^−^ is converted into peroxynitrite (ONOO^−^) and hydroxyl radical (OH^∙^) through a reaction with nitric oxide (NO) [29]. OH^∙^ is generated by a ferrous iron-mediated reduction of H_2_O_2_ and the decomposition of ONOO^−^ [29]. Additionally, H_2_O_2_ can be converted into hypochlorous acid and hypobromous acid (HOCl and HOBr) through myeloperoxidase in the phagocytic vacuole in neutrophils for pathogen defense [22, 30, 31] (Figure 3). Meanwhile, biologically relevant ROS are also derived from the exogenous environment, which includes air pollutants, stress, ultraviolet rays, toxicants, tumor chemotherapy, and radiotherapy [24, 32–35]. However, these exposures are highly variable; it is challenging to measure ROS directly in cells and tissues.

### 2.2. The Impact and Damage Outcomes of ROS

Among the radical and nonradical oxygen species, H_2_O_2_ is recognized as the key redox signaling agent in redox regulation of biological activities, and a total of 37 H_2_O_2_­generating enzymes have been found [36, 37]. It is now clear that H_2_O_2_ plays a fundamental role in physiology as a functional signaling entity [38]. H_2_O_2_ first occurred at low homeostasis levels in normally breathing eukaryotic cells, and it was the primary ROS responsible for protein oxidation [39]. Generally, the generation of H_2_O_2_ was constantly stimulated by metabolic cues or various stressors intracellularly, and the concentration of H_2_O_2_ is maintained in the low nanomolar range, which is important for signaling by redox signaling via oxidation and called “oxidative eustress” [40, 41]. The overall cellular concentration of the O_2_^−^ is maintained at about 10^–11^ M, which is much lower than the 10^–8^ M of H_2_O_2_ [42]. Diffusible H_2_O_2_ contributes to orchestration of various processes including cell proliferation, differentiation, and angiogenesis through oxidation of sulfur (thiolate groups) in target proteins and further activates stress responsive survival pathways [43, 44]. Meanwhile, H_2_O_2_ acts as signal transduction molecules that induce proinflammatory cytokines and the nuclear factor-*κ*B (NF-*κ*B) pathway [45, 46].

In contrast to low levels of H_2_O_2_, supraphysiological concentrations of H_2_O_2_ cause “oxidative distress,” which can induce a plethora of irreversible damaging effects to proteins, DNA, and lipids and ultimately cause cell death [47]. At the cellular level, oxidation of proteins by ROS is more common than that of DNA and lipids [48]. When proteins are exposed to ROS, amino acid side chains are modified, and consequently, the protein structure is altered [48]. ROS can cleave peptide bonds through *α*-amidation, diamidation, proline residue oxidation, glutamine residue oxidation, and aspartyl residue oxidation [49]. ROS-induced protein oxidation may contribute to the following: (1) hydroxylation of aromatic groups and aliphatic amino acid side chains, nitration of aromatic amino acid residues, nitrosation of sulfhydryl groups, and sulfonation of methionine residues; (2) polypeptide chain breaking to form cross-linked protein aggregates; and (3) the functional groups of proteins reacting with oxidation products of polyunsaturated fatty acids or carbohydrate derivatives, which affects normal physiological function [48, 49]. It is equally well known that sustained exposure to high ROS levels can damage DNA through single strand break, point mutations, miscoding, and abnormal amplification [48]. DNA is complexed as chromatin with histones; ROS can further affect the oxidation and reduction of adduct radicals of DNA [48]. Besides, toxic concentrations of ROS also induce mitochondrial DNA mutations [48, 50]. Lipids have the functions of energy storage, signal transduction, transport, and cell membrane composition in cells, and many types of lipids are easily oxidized by ROS [48, 51]. The reaction of ROS with lipid molecules can activate the lipid peroxidation free radical cascade, which is generally very fast [48]. The hydrogen atom abstraction forms a methylene carbon of a polyunsaturated fatty acid by a lipid hydroperoxyl radical, forming a new carbon centered radical that propagates the peroxidative chain reaction and a hydroperoxide [52]. Moreover, the more double bonds in the lipid, the easier it is for hydrogen atoms to be taken away [53]. Excessive ROS can cause lipid peroxidation in biofilms, which would result in loss of fluidity, abnormal membrane potential, and rupture and leakage of cell contents [54]. Therefore, it is challenging and important to determine the precise role and maintain a safe cellular ROS gradient and regulate redox signaling pathways.

### 2.3. Intracellular Clearance of ROS

Excess ROS production induces a plethora of damaging effects to cellular biomacromolecules. Hence, supraphysiological gradients of ROS are showcased as harmful species, and buffering ROS to maintain redox homeostasis is required. In order to prevent the unrestricted accumulation of ROS, a series of antioxidant defense systems have been discovered and can act independently or synergistically to neutralize ROS. Antioxidants can be divided into two groups, that is, noncatalytic small molecules and catalytic antioxidants [29]. Glutathione (GSH) is the most abundant nonenzymatic antioxidant molecule and is essential for cell survival and redox homeostasis [55]. GSH is a tripeptide that synthesis catalyzed by glutamate-cysteine ligase (GCL) and GSH synthetase (GSS), and it is used as a cofactor by GSH S-transferases (GSTs) and GSH peroxidases (GPXs) to eliminate ROS [56]. Besides, endogenously synthesized bilirubin, melatonin, *α*-lipoic acid, and uric acid are other nonenzymatic antioxidant molecules that mitigate the excess level of ROS produced in cells [29, 57]. Enzymatic antioxidants include SOD, catalase (CAT), peroxiredoxins (PRXs, also called PRXs), glutathione peroxidases (GPxs), thioredoxin reductases (TrxRs), and thioredoxins (Trxs) [29]. Enzymatic antioxidants with high catalytic activity are uncovered as handling ROS levels in cells [29]. SODs are a family of metalloenzymes catalyzing the dismutation of O_2_^−^ to H_2_O_2_, which utilizes metal ions, including copper (Cu^2+^), ferrous iron (Fe^2+^), manganese (Mn^2+^), and zinc (Zn^2+^) as cofactors [58]. CAT is primarily localized in the cytosol and cell organelles called the peroxisome, which can convert H_2_O_2_ into O_2_ and H_2_O [59]. In addition, Trxs promote PRDX-mediated H_2_O_2_ detoxification and reduction of lipid by GPx requires GSH [56]. More importantly, GSH and Trxs generate oxidized forms through detoxification of ROS [56]. Oxidized GSH and Trxs are both regenerated by GSH reductase (GSR) and Trxs reductase 1 using NADPH as a cofactor, respectively [60]. GSH and Trxs, and TrxRs are noncatalytic and catalytic antioxidants, which are critically involved in different stages of cancers [61–63].

In addition, it is established that many transcription factors, including nuclear factor erythroid 2-related factor 2 (NRF2), the forkhead box O (FOXO), hypoxia-inducible factor (HIF), NF­*κ*B, and tumor protein p53 (TP53 or Trp53 in mice), are activated by ROS and regulate intracellular redox environment of cells [64]. NRF2 is the most important transcription factor for the activation of a number of genes that have antioxidant functions within the cell [65, 66]. However, under resting conditions, NRF2 is degraded through interacting with Kelch-like ECH-associated protein 1- (KEAP1-) Cullin 3 (CUL3) E3 ligase complex. Under conditions of oxidative stress or electrophilic addition, cysteine residues on KEAP1 are modified, thus blocking NRF2 interaction and subsequent degradation [67]. Then, NRF2 translocated into the nucleus, where it serves as a transcription factor for expression of the antioxidant responsive element- (ARE-) driven genes, including hemeoxygenase-1 (HO-1), NAD(P)H quinone oxidoreductase 1 (NQO1), glutathione S-transferases (GSTs), and UDP-glucuronosyltransferases (UGTs) [67]. In addition, sestrins (SESN1, 2, and 3) exert indirect antioxidant activity, in part by activation of transcription factor NRF2 [68, 69]. The FOXO family of transcription factors contributes to the maintenance of cellular and organismal homeostasis in various ways [70]. For example, FOXO improves mitochondrial redox, suppresses the levels of free transition metal ions, and promotes antioxidant defense system [71]. Hypoxia has been associated with an increase in O_2_^−^ and H_2_O_2_ generation through inhibition of the mitochondrial ETC [72]. HIF is a transcription factor that serves as the master regulator of transcriptional responses to hypoxia [73]. Oxidants can stabilize HIF during hypoxia, thereby helping to increase the hypoxia response [73]. NF-*κ*B serves as a master switch of inflammation, which is associated with extensive H_2_O_2_ production [74]. In different context, H_2_O_2_ has different roles in NF­*κ*B function [74]. H_2_O_2_ activates NF­*κ*B pathway and then negatively controls the stability of I*κ*B in the cytosol [75], while H_2_O_2_ also directly modulates NF­*κ*B due to the presence of oxidizable cysteines in the DNA-binding region of NF-*κ*B [76]. The tumor suppressor protein p53 was considers the transcription factor that has a major role in regulating antioxidant gene expression [77, 78]. Under the control of H_2_O_2_, it regulates the selective transduction activation of p53 target genes through the oxidation of p53 cysteine residues. Reciprocally, p53 regulates the expression of antioxidant genes to maintain cellular redox balance [79]. Other transcription factors, such as AMP-activated protein kinase (AMPK), activator protein 1 (AP-1), heat shock factor 1 (HSF1), peroxisome proliferator-activated receptor *γ* coactivator-1*α* (PGC-1*α*), uncoupling protein (UCP), and protein­tyrosine phosphatase 1B (PTP1B), also contribute to redox status [80–85].

**Figure 4:**
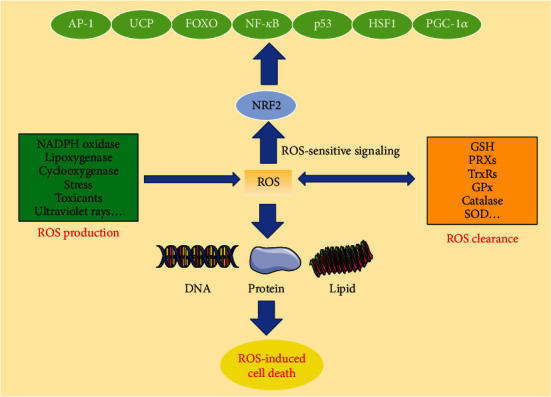
ROS balance and their roles in regulating transcription factors and cell death. ROS can be produced by NADPH oxidases, lipoxygenase, cyclooxygenase, stress, toxicants, and ultraviolet rays. On the other hand, ROS can be eliminated via activation of the GSH, PRXs, TrxRs, GPx, catalase, and SOD. Extremely high levels of ROS are dangerous for the DNA, protein, and lipid and eventually cause cell death. Cells first adapt to the increase in ROS by activating NRF2 and then trigger other members of the antioxidant transcription factor when the excess levels of ROS that are not countered by the NRF2-directed defenses.

However, the extents to which individual members of the above network of antioxidant transcription factors are differentially activated by oxidative stress are uncertain, although it is improbable that all of them are activated simultaneously. However, different transcription factors may respond to distinct threshold levels of ROS. When cells suffered from moderate levels of ROS, NRF2 first was activated, and then a series of genes encoding detoxification enzymes were further induced, which provided a floodgate to protect against ROS [20]. When cells further adapt to sustained exposure to high ROS levels, which causes activation of Krüppel-like transcription factor 9 (KLF9) and downregulation of NRF2, the NRF2-induced defense cannot counteract the excess ROS, then triggering additional redox switches that activate other members of the antioxidant transcription factor network [20] (Figure 4). Therefore, intracellular ROS regulation is closely related to the above complex processes, and there is no constant boundary between prooxidants and antioxidants in the regulation of ROS.

## 3. ROS Paradox and Contradictory Strategies Based on ROS for Cancer Treatment

**Figure 5:**
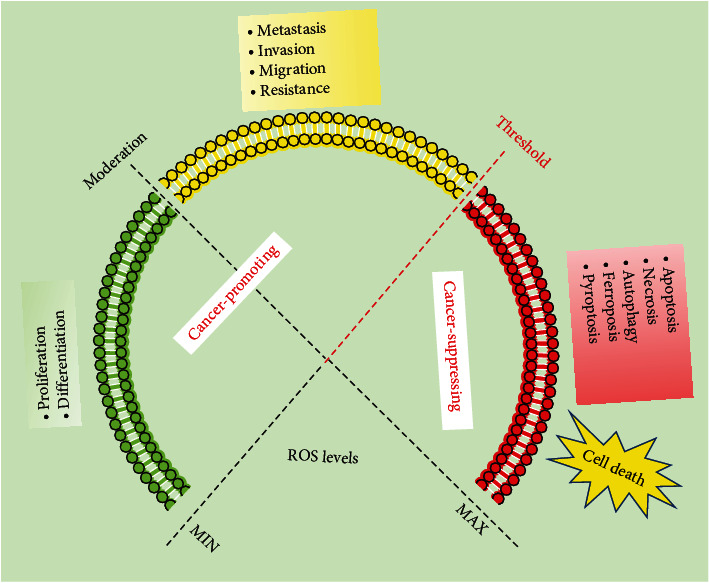
The cancer-promoting activities and cancer-suppressing activities of ROS in cancer. Low ROS (green) is the basic need to maintain normal cellular proliferation, and differentiation. Moderate ROS (yellow) is the signal for the increased cellular metastasis, invasion and migration, and resistance. When the ROS level exceeds threshold (red), ROS can induce cancer cell death via the activation of cell apoptosis, necrosis, autophagy, ferroptosis, and pyroptosis.

Under normal physiological conditions, the redox system is in good coordination and well-balanced. However, in the presence of obvious stimuli, the balance would be disrupted, triggering oxidative stress and in turn increasing ROS levels, implicated in various human diseases including cancer. Interestingly, oxidative stress can activate cell survival or death mechanisms depending on the severity and exposure time of ROS excess. In general, ROS act as mitogens to induce proliferation and differentiation of normal and cancer cells at low concentrations (usually submicromolar concentrations) [48]. At moderate concentrations, ROS have been implicated in tumor initiation and progression, malignant conversion, and resistance to chemotherapy. The higher concentrations of ROS result in damage cellular biomolecules and cause gene mutations, thus promoting canceration of normal cells or inducing cancer cell apoptosis, necrosis, autophagy, ferroptosis, and pyroptosis [48, 86] (Figure 5). Therefore, the roles of ROS are complicated, and ROS operate as a diversified biochemical entity in cancer progression.

Because the influence of ROS on cancer development is contradictory, reducing or increasing intracellular ROS levels would be a potential strategy to prevent or treat cancer [87]. Namely, reducing the intracellular ROS content by inhibiting ROS production pathway and using exogenous supplementation of antioxidants is an effective strategy, and it could effectively prevent the early stage of tumor occurrence. Cancer cells are more sensitive to enhanced intracellular ROS than normal cells; thus, cancer cells can be preferentially killed by enhancing the cellular ROS levels, which might be another puissant strategy to selectively kill cancer cells. Moreover, the expression level of antioxidant enzymes and oxidative stress environment in drug-resistant tumor cells are usually higher; ROS-modulating drugs may have a better therapeutic effect on the intervention of drug-resistant tumor cells. The use of small molecules to increase the production of ROS or/and inhibit the antioxidant defense system is one of the most effective anticancer methods [87]. In recent years, several clinical trials have been made in the research of therapeutic drugs targeting ROS regulation in cancer cells [88]. Moreover, small molecules regulating ROS homeostasis for cancer therapy have been comprehensively reviewed [24, 87]. However, most small molecules described in the literature or on the clinical development stages have not entered into clinical treatment for cancer. Several FDA-approved drugs based on ROS regulation have led to repurposing of cancer indications, which may be considered a novel and valid cancer therapeutics [89]. Therefore, in this paper, we will focus on repurposed drugs for cancer therapy by the regulation of ROS homeostasis.

## 4. Repurposed Drug-Regulated ROS Homeostasis as a Novel Cancer Therapeutics in Oncology

### 4.1. Repurposed Drugs as a ROS Scavenger in Cancer

ROS accumulation is one of the initiating factors in the early stage of the neoplastic process. To this extent, ROS can lead to more metabolic adaptations and more levels of DNA damage and genetic instability in normal cells, consequently promoting the cancer cell proliferation and growth [90, 91]. Numerous epidemiological data and preclinical/clinical studies suggest that small molecule ROS inhibitors can effectively prevent tumorigenesis. Therefore, repurposed drugs that act as ROS scavengers have the potential to modulate levels of ROS for therapeutic benefit in cancer.

#### 4.1.1. Vitamin C

Vitamin C, known as ascorbic acid, is an antioxidant converted from glucose, which is abundant in fresh fruits and vegetables [92]. At physiological concentrations, vitamin C prevents gene mutations caused by peroxidation by removing ROS, and it also blocks oxidative modification of amino acids to maintain protein integrity and protects lipids from peroxidation [93]. In a cohort study, Wright et al. analyzed the comprehensive intake of individual selenium, flavonoids, vitamin C, and carotenoids to predict the risk of lung cancer. They proved that integration of dietary antioxidants can significantly reduce lung cancer incidence in male smokers [94]. However, it has been nearly half of a century since the beginning of researches of anticancer mechanism of vitamin C, and its role was challenged and verified repeatedly. Moreover, the controversy about the anticancer efficiency of vitamin C may depend on the administration ways (oral or intravenous), which can result in different concentrations in the plasma of cancer subjects [92]. High-dose vitamin C alone or in combination can inhibit tumor growth in various cancer models through regulation the level of ROS [95–97]. Furthermore, high-dose intravenous vitamin C in cancer patients has led to increased quality of life with minimal side effects [98, 99]. Several excellent reviews have recently described that vitamin C is used for cancer chemoprevention and clarified that the anticancer mechanism of high doses of vitamin C is targeting excessive ROS generation and/or epigenetic regulators and/or hypoxia-inducible factor 1 (HIF-1) [92, 100, 101]. Moreover, vitamin C has also been extensively tested in clinical trials of cancer for many years (Table 1).

#### 4.1.2. Vitamin E

Vitamin E is a hydrophobic fat-soluble compound that exists in a variety of food sources, and it in nature occurs as 8 isoforms (tocopherols and tocotrienols, both as *α*, *β*, *γ*, and *δ* forms); however, only *α*-tocopherol is considered to be essential for human [102, 103]. Vitamin E protects cells from cell damage caused by ROS, thereby attenuating DNA damage and cancer development [102]. Vitamin E has been extensively studied, and much data indicates that it has a role in cancer prevention [103]. For example, it is found that vitamin E can interfere phosphotidylinositol-3-kinase/protein kinase B (PI3K/PKB) and protein kinase C (PKC) signaling pathways by scavenging ROS, which may be one of the antitumor mechanisms [102, 104–106]. Moreover, clinical trials of vitamin E in cancer treatment have been detailed in some review and research articles [107, 108], although it was found that the development and metastasis of lung tumors were increased in vitamin E-treated mouse models [109].

### 4.2. Repurposed Drugs as a ROS Inducer in Cancer

Interestingly, repurposed drugs are more likely to act as ROS inducers in the treatment or prevention of cancer. ROS-inducing repurposed drugs by mechanisms of inhibiting intracellular antioxidant systems and/or producing ROS generation in cells were reported to selectively kill cancer phenotypes. Several reviews on the small molecules regulating ROS homeostasis for cancer therapy have been published [24, 87, 89]. In the review, we will classify and describe repurposed drugs that induce the excessive production of ROS in the cancer cells to exert antitumor effects.

#### 4.2.1. Antibacterial

*(1) Tigecycline*. Tigecycline is a broad-spectrum antibiotic approved by the FDA for the treatment of multidrug-resistant bacterial infections, complicated intra-abdominal infections, complicated skin structure infections, and community-acquired pneumonia [110]. Its antibacterial mechanism involves killing bacteria by binding to the 30S bacterial ribosomal subunit, thereby preventing tRNA and its codons from linking to the A site of the ribosomal complex, which result in inhibiting protein synthesis [111, 112]. Recent studies have found that tigecycline is identified as one of the effective anticancer agents by enhancing the levels ROS. For example, tigecycline inhibited mitochondrial respiration, mitochondrial membrane potential, and adenosine triphosphate (ATP) levels and caused an increase in intracellular ROS in a dose-dependent manner, which induced death of non-small-cell lung cancer cells [113]. Additionally, tigecycline was found to selectively kill leukemic stem and progenitor cells by inhibiting mitochondrial translation [114]. Besides, tigecycline significantly enhanced conventional cisplatin activity against human hepatocellular carcinoma through inducing mitochondrial dysfunction and increasing the levels of mitochondrial superoxide, hydrogen peroxide, and ROS levels [115]. More importantly, a phase I clinical trial evaluating the safety and biologic activity of intravenous infusions of tigecycline to treat acute myeloid leukemia was completed (NCT01332786) (Table 1).

*(2) Levofloxacin*. Levofloxacin is a third-generation fluoroquinolone antibacterial drug, which can kill bacterial through preventing DNA replication [116]. It is often used clinically for some moderate and severe infections caused by sensitive bacteria [116]. With the further study, repurposed antibiotic levofloxacin is an attractive candidate for cancer treatment. It was found that levofloxacin effectively inhibited lung cancer cell proliferation and induces apoptosis [117]. Mechanistically, levofloxacin inhibited the activity of the mitochondrial electron transport chain complex, which in turn blocked mitochondrial respiration, reduced ATP production, and increased the levels of ROS, mitochondrial superoxide, and hydrogen peroxide [117]. Moreover, levofloxacin effectively targeted breast cancer cells and acted synergistically with 5-fluorouracil through inhibiting mitochondrial biogenesis and was accompanied by the deactivation of PI3K/PKB/mammalian target of rapamycin (mTOR) and mitogen-activated protein kinase/extracellular signal-regulated kinase (MAPK/ERK) signaling pathways [118].

*(3) Doxycycline*. Doxycycline (DOXY), a derivative of tetracycline, is a broad-spectrum antibiotic that exhibits many therapeutic activities in addition to its antibacterial properties [116, 119]. Doxycycline has recently carved out a role in cancer therapy. It was found that doxycycline triggered cell death in different cancer cells, including cervical, breast, lung, and prostate cancer cells [120]. A further study found that doxycycline was effective in targeting glioblastoma through inducing mitochondrial dysfunctions and oxidative stress [121]. Moreover, the ROS-apoptosis signal regulating kinase 1- (ASK1-) Jun N-terminal kinase (JNK) pathway is involved in doxycycline-induced melanoma cell death [122]. Amplification of tumor-associated ROS has been used as a boosting strategy to improve tumor therapy. A recent study has shown that prodrug chlorin e6 (Ce6) and zoledronic acid (ZA)/mesoporous silica nanoparticles (MSN)/doxorubicin- (DOX-) thioketal- (TK-) DOXY can be used for the chemodynamic therapy of osteosarcoma [123]. Upon laser irradiation, the loaded Ce6 produced in situ ROS and subsequently resulted in DOX/DOXY release. The released DOXY promoted ROS production and further induced ROS burst, which increased the sensitivity of the osteosarcoma to chemotherapy and resulted in enhancing tumor cell inhibition and apoptosis [123]. Furthermore, some clinical trials are ongoing, including a phase II trial study of how well metformin hydrochloride works together with doxycycline in treating patients with localized breast or uterine cancer (NCT02874430) and a study of doxycycline for the treatment of cutaneous T-cell lymphoma (NCT02341209) (Table 1).

*(4) Clarithromycin*. Clarithromycin belongs to a family of 14-membered ring macrolide antibiotics, but several clinical investigations showed that clarithromycin was highly efficient for multiple myeloma (MM) when used in combination with conventional chemotherapy since 1997 [124]. This finding highlights the importance of clarithromycin on the treatment of MM and offers a new regimen for the relapsed/refractory MM patients. Moreover, the results of Zhou et al. showed that clarithromycin plus cisplatin had a synergetic effect against ovarian cancer cell viability and induced the apoptosis rate, which was linked to the increase of ROS levels *in vitro* and *in vivo* [125]. This result proved that clarithromycin augmented cisplatin response via a ROS-mediated synergistic effect. However, no clinical trials have investigated the activity of clarithromycin against ovarian cancer. Indeed, a phase II clinical trial evaluating clarithromycin treatment for cachexia (the loss of muscle mass) in people with non-small-cell lung cancer was terminated due to having not enough participants (NCT02416570) (Table 1).

#### 4.2.2. Anthelmintic

*(1) Niclosamide*. Niclosamide, an FDA-approved oral agent, belongs to the antiparasitic disease drug and has been used in the clinical treatment of intestinal parasitic infections for nearly 50 years [126]. In recent studies, it had been documented that niclosamide had antitumor effects and can increase the sensitivity of tumor cells to chemotherapy and radiotherapy through regulating redox homeostasis. For example, niclosamide inhibited the NF-*κ*B pathway and increased ROS levels to induce apoptosis in acute myelogenous leukemia cells [127]. Niclosamide also suppressed renal cell carcinoma by inhibiting Wnt/beta-catenin and inducing mitochondrial dysfunctions [128]. Moreover, niclosamide was found to sensitize the responsiveness of cervical cancer cells to paclitaxel via ROS-mediated mTOR inhibition [129]. Also, niclosamide was chosen based on a cell-based high-throughput viability screen and it had a radiosensitizing effect on H1299 human lung cancer cells [130]. A further study had demonstrated that niclosamide plus gamma-ionizing radiation can produce ROS and promote c-Jun and its phosphorylation [130]. Moreover, niclosamide also acted as a potent radiosensitizer through inhibiting signal transducer and activator of transcription 3 (STAT3) and B-cell lymphoma-2 (Bcl-2) and increasing ROS generation in triple-negative breast cancer cells [131]. The therapeutic effect of combination valproic acid and niclosamide was investigated on human lung cancer cell line [132]. The results showed that combination therapy caused a dramatic decrease in cell viability by inducing the extrinsic apoptotic pathway and stimulating endoplasmic reticulum stress and mitochondrial membrane potential loss associated with increased ROS levels [132]. Based on these encouraging results, the evaluation of niclosamide in several clinical trials has been investigated (Table 1).

*(2) Albendazole*. Albendazole is a broad-spectrum, low-toxic antiparasitic drug that kills susceptible parasites by reducing the glycogen stores and the formation of ATP [133]. There are several evidences supporting albendazole repositioning for cancer therapy against tumor cell lines [133]. Further studies have shown that oxidative stress was one of anticancer mechanisms that mediated albendazole. Castro et al. had demonstrated that albendazole treatment could trigger apoptosis and induce MCF-7 cell death through ROS generation, which was related to depletion of reduced glutathione levels, augmented important oxidative biomarkers, and increased the activity of antioxidant enzymes [134]. It was found that ROS can induce p38 MAPK activation in U937 cells treated with albendazole. Pretreatment with SB202190 (p38 MAPK inhibitor) increased the activity of cells treated with albendazole, indicating that ROS-induced P38 MAPK activation was associated with albendazole-mediated cell death [135]. However, no clinical trials have been conducted to investigate the antitumor effects of the albendazole.

#### 4.2.3. Antimalarial

*(1) Artemisinin*. Artemisinin and its derivatives are natural synthetic antimalarial drugs [136]. With the deepening of research, artemisinin not only has strong antimalarial activity but also has obvious antitumor effects. Artemisinin harbors an endoperoxide bridge whose cleavage results in the generation of ROS and/or artemisinin carbon-centered free radicals, further promoting cell apoptosis, inhibiting cell proliferation and damaging DNA, cell membrane, protein, and organelles to play an antitumor effect [137]. The ROS-mediated antitumor properties of artemisinin on numerous cancer types have been reported [138–142]. Compared with traditional chemotherapeutic drugs, artemisinin has the advantages of broad antitumor spectrum, less toxicity, and side effects, so it can be identified as an intriguing candidate for repurposing. However, there are no clinical trials investigating the antiproliferative effects of artemisinin; an additional study is necessary for optimal clinical efficacy.

*(2) Hydroxychloroquine*. Hydroxychloroquine, a chloroquine derivative, is originally developed to treat patients with malaria, but it has been further investigated because of its antiproliferative effects on different types of tumors [143]. In general, hydroxychloroquine has a better oral bioavailability and safety profile than chloroquine, which makes it a suitable candidate to evaluate its potential therapeutic applications in cancer [143]. Hence, hydroxychloroquine was under investigation in cell level, animal models, and clinical trials for a variety of cancers. Many studies found that hydroxychloroquine was capable of killing tumor cells by different pathways accompanied by the massive production of ROS. In-depth evaluation of hydroxychloroquine, it revealed that it could be considered an effective autophagy inhibitor [144]. Autophagy is a self-degrading intracellular process involving tumor suppression and promotion [145]. However, inhibition of autophagy with hydroxychloroquine can not only hinder the autophagic protective effect but also increase dysfunctional mitochondria and ROS production, and a further study found that ROS was the main mechanism of enhanced cytotoxicity with autophagy inhibition [144]. Moreover, hydroxychloroquine exhibited a good synergism with microtubule polymerization inhibitor CYT997 on the induction of ROS-associated apoptosis in human head and neck squamous cell carcinoma [146]. In addition, breast cancer cell apoptosis induced by hydroxychloroquine was related to the inhibition of the autophagic flux and accumulation of damaged mitochondria and ROS [147]. Hence, the inhibition of autophagy is, at least partially, responsible for hydroxychloroquine-mediated upregulation of ROS in cancer cell death.

#### 4.2.4. Cardiovascular

*(1) Simvastatin*. Simvastatin is an antihigh cholesterol drug widely used in the prevention and treatment of cardiovascular diseases by inhibiting the 3-hydroxy-3-methylglutaryl-coenzyme a (Hmg-CoA) reductase in the mevalonate pathway and blocking the formation of intermediary products in the biosynthesis of cholesterol [148]. Simvastatin has recently been considered a potential sensitizer to chemotherapy and radiotherapy and exhibits inhibitory effects on amounts of types of cancer [149]. For example, simvastatin alone or in combination with doxorubicin significantly increased ROS levels and suppressed breast cancer MCF-7 cell proliferation [150]. Moreover, a combined therapy of simvastatin and pentoxifylline effectively activated ERK/AKT, upregulated ROS levels, downregulated p-p38, and inhibited NF-*κ*B signaling pathway, thereby promoting triple-negative breast cancer cell apoptosis [150]. Additionally, simvastatin administration alone also could induce ROS formation in the KKU-100 cells [151]. Due to the excellent antitumor effect of simvastatin *in vitro*, a large number of clinical studies have been conducted (Table 1).

*(2) Digoxin*. Digoxin, an inhibitor of Na^+^/K^+^ ATPase, is widely used to treat heart failure. The clinical tests of digoxin as an anticancer drug, alone or in combination with chemotherapeutic drug, were reported [152]. Anticancer effects of digoxin involve various mechanisms. For example, Wang et al. reported that digoxin inhibited p53 synthesis by activating Src/MAPK signaling pathways and suppresses tumor growth [153]. In addition, digoxin induced apoptosis and cell cycle arrest and had antitumor effects on Burkitt lymphoma cells *in vitro* and *in vivo* [154]. Many studies also reported that digoxin promoted ROS generation via inhibiting hypoxia-inducible factor-1alpha (HIF-1*α*), a key regulator of angiogenesis, to block cell growth in a multiple tumor model [155–157]. Moreover, digoxin was found to inhibit activity of the NRF2-ARE luciferase reporter gene in A549-ARE cells, which suggested that digoxin may be a potent NRF2 inhibitor [158]. Zhou et al. found that digoxin could reverse drug resistance of gemcitabine in SW1990/Gem and Panc-1/Gem cells [159]. Mechanistically, digoxin inhibited the activity of NRF2 by suppressing PI3K/Akt signaling pathway in gemcitabine-resistant pancreatic cancer cells [159]. To date, digoxin has been investigated in clinical trials for cancer therapy (Table 1).

#### 4.2.5. Antipsychotics

*(1) Fluphenazine*. Fluphenazine is a phenothiazine antipsychotic drug, which is an antagonist of dopamine D1 and D2 receptors and has a high affinity with 5-HT receptors [160]. It is used in the treatment of schizophrenia and bipolar disorder [160]. Studies have shown an overall decreased cancer incidence in schizophrenic patients using antipsychotics, implying that antipsychotics may have anticancer potentials [161]. As expected, research found that fluphenazine may play an important role in the treatment of cancer [161]. It was found that the ROS levels in triple negative breast cancer cells were significantly increased after fluphenazine treatment, which could impair the mitochondria membrane integrity and further induce cancer cell death [162]. Moreover, HeLa cancer cells treated with fluphenazine in combination with UVA light demonstrated a consistent ROS production in a clearly concentration-dependent manner, indicating a significant photodynamic mechanism involved in the photocytotoxic effect of fluphenazine [163]. Moreover, a clinical trial of fluphenazine in treating patients with refractory advanced multiple myeloma was completed (NCT00335647) (Table 1).

*(2) Pimozide*. Pimozide is an FDA-approved antipsychotic, and it is used to treat clinical Tourette syndrome and schizophrenia [164]. In 1979, pimozide was first found to act as a dopamine antagonist with antimelanoma cancer effect [165]. After that, pimozide had been investigated in a number of cancer cells, and a further study found that pimozide inhibited the cancer cells through the generation of ROS [166]. For example, pimozide induced ROS generation by downregulating the expression of the antioxidant enzyme catalase to suppress osteosarcoma and prostate cancer [166, 167]. Moreover, recently, the ability of ROS generation to suppress hepatocellular carcinoma cells has been reported [168]. However, to date, pimozide has not been investigated in clinical trials to clarify the antitumor activity.

## 5. Research Perspectives and Discussion

Despite the fact that traditional approaches of looking for differences in the transcriptome or the proteome in cancer have many benefits, much attention has been focused on significant changes in function, such as regulating ROS level, which may be an effective anticancer strategy [169, 170]. Certainly, many clinical chemotherapeutic drugs, such as doxorubicin, daunorubicin, and epirubicin, can kill cancer cells by enhancing ROS production. However, the uses of these drugs are accompanied by indiscriminate cytotoxicity and adverse events and chemoresistance. Repurposed drugs with established safety profiles that are developed based on clearing ROS generation or increasing ROS production may be a novel strategy for the treatment of cancers. However, repurposed drugs may be the lack of specificity for cancer. Moreover, ROS are considered a double-edged sword in cancer. The molecular action of ROS is multidirectional, which in turn produces many uncertainties. There are still some key issues that need to be resolved in the development of ROS-related repurposed drugs.

It is necessary to first understand whether repurposed drugs based on ROS regulation can really be used clinically to treat cancer. The benefits of antioxidant drugs for early cancer therapies by reducing ROS level have been widely recognized. However, several studies have demonstrated antioxidant drugs produced the paradoxical results. Long-term supplementation with the antioxidants N-acetylcysteine and vitamin E promotes KRAS-driven lung cancer metastasis [109]. In addition, it has been shown that the administration of antioxidants, such as N-acetylcysteine, accelerates the progression of lung cancers and melanomas [171]. Thus, whether antioxidants inhibit or promote tumors needs further support by solid trials performed on a large scale. Meanwhile, raising ROS to cytotoxic levels can kill cancer cells; this strategy may inevitably damage normal cells. Since the dose of chemotherapy drugs clinically is much higher than the dose required for the original effect of the repurposed drugs, it may be difficult to obtain an effective and safe dose clinically. Repurposed drugs may produce ultrahigh ROS levels that the human body cannot tolerate when administered rapidly and at a high concentration, which can significantly induce systemic toxicity to cancer patients. In this regard, ROS-related repurposed drugs are more suitable for use as chemotherapy sensitizers or adjuvant drugs in tumor treatment, which may lead to unexpected response. Moreover, further elucidation of ROS-related cysteine modifications and their functional consequences will be the basis for improving our understanding of the selective effects of ROS on cancer and normal cells.

Another major challenge is increasing the selectivity of ROS-related repurposed drugs as therapeutic drugs. Cancer cells thrive on levels of ROS that are moderately higher than those in their normal counterparts; this feature renders ROS-responsive photodynamic therapy can reach good results. Up to now, new ROS-responsive prodrugs, probes, theranostic prodrugs, and nanotheranostics that allow for the monitoring of ROS with temporal and spatial specificity have been developed for the targeted treatment and precise diagnosis of cancer and selectively killing tumor cells [172, 173]. In fact, ROS-responsive prodrug strategies have been successfully used to modify clinically platinum-based drugs, showing enhanced therapeutic efficacy and reduced side effects [174, 175]. Therefore, the development of repurposed drugs inspired ROS-responsive groups/probes/nanoparticles would be a significant improvement in cancer treatment selectively.

In future research, before adopting the treatment method, there should be advanced inspection and real-time monitoring of the ROS status in the body, and ROS-related repurposed drugs should be taken appropriately to increase or decrease the ROS level in the body, so as to obtain a better treatment effect.

## Figures and Tables

**Table 1:** Repurposed clinical candidates for cancer.

Drug	Original indication	Clinical trials
Vitamin C	Antioxidant	(1) Effect of vitamin C and E in breast cancer patients undergoing chemotherapy (nct04463459) (2) Preoperative IMRT with concurrent high-dose vitamin C and mFOLFOX6 in locally advanced rectal cancer (nct04801511) (3) Intravenous (IV) vitamin C with chemotherapy for cisplatin-ineligible bladder cancer patients (nct04046094) (4) Intravenous ascorbic acid supplementation in neoadjuvant chemotherapy for breast cancer (nct03175341) (5) Ph 2 trial of vitamin C & G-FLIP (low doses of gemcitabine, 5FU, leucovorin, irinotecan, and oxaliplatin) for pancreatic cancer (nct01905150) (6) Other clinical trials are available at clinicaltrials.gov
Vitamin E	Antioxidant	(1) Vitamin E supplements in preventing cancer in patients at risk of prostate cancer or who have prostate cancer (nct00895115) (2) Vitamin E supplements in treating patients undergoing surgery for colorectal cancer (nct00905918) (3) Selenium and vitamin E in preventing cancer progression and recurrence in patients with early-stage bladder cancer (nct00553345) (4) S0000 selenium and vitamin E in preventing prostate cancer (nct00006392) (5) A pilot clinical trial with tocotrienol on breast cancer (nct01157026) (6) Other clinical trials are available at clinicaltrials.gov
Tigecycline	Antibacterial	(1) Personalized treatment of urogenital cancers depends on the microbiome (nct03962920) (2) Safety study evaluating intravenous infusions of tigecycline to treat acute myeloid leukemia (nct01332786) (3) In vitro study of tigecycline to treat chronic myeloid leukemia (nct02883036)
Doxycycline	Antibacterial	(1) Metformin hydrochloride and doxycycline in treating patients with localized breast or uterine cancer (nct02874430) (2) Doxycycline for the treatment of cutaneous T-cell lymphoma (nct02341209) (3) Combining doxycycline with bone-targeted therapy in patients with metastatic breast cancer (nct01847976) (4) Doxycycline in lymphangioleiomyomatosis (lam) (nct00989742) (5) Doxycycline, temozolomide, and ipilimumab in melanoma (nct01590082) (6) A phase II study of doxycycline in relapsed NHL (nct02086591)
Clarithromycin	Antibacterial	(1) Is clarithromycin a potential treatment for cachexia in people with lung cancer? (nct02416570) (2) A trial with metronomic low-dose treosulfan, pioglitazone, and clarithromycin versus standard treatment in NSCLC (nct02852083) (3) Chemoprevention therapy in treating patients at high risk of developing multiple myeloma (nct00006219) (4) Clarithromycin in multiple myeloma induction therapy (nct02573935) (5) Clinical trial of clarithromycin, lenalidomide, and dexamethasone in the treatment of the first relapsed multiple myeloma (nct04063189) (6) Other clinical trials are available at clinicaltrials.gov
Niclosamide	Antiparasitic	(1) A study of niclosamide in patients with resectable colon cancer (nct02687009) (2) Drug trial to investigate the safety and efficacy of niclosamide tablets in patients with metastases of a colorectal cancer progressing after therapy (nct02519582) (3) Enzalutamide and niclosamide in treating patients with recurrent or metastatic castration-resistant prostate cancer (nct03123978) (4) Niclosamide and enzalutamide in treating patients with castration-resistant, metastatic prostate cancer (nct02532114) (5) Abiraterone acetate, niclosamide, and prednisone in treating patients with hormone-resistant prostate cancer (nct02807805)
Hydroxychloroquine	Antimalarial	(1) Phase I/II study of hydroxychloroquine with itraconazole with biochemically recurrent prostate cancer (nct03513211) (2) Hydroxychloroquine in previously treated patients with metastatic pancreatic cancer (nct01273805) (3) Hydroxychloroquine to increase tumor suppressor par-4 levels in oligometastatic prostate cancer (nct04011410) (4) Hydroxychloroquine in metastatic estrogen receptor-positive breast cancer progressing on hormonal therapy (nct02414776) (5) Hydroxychloroquine and gefitinib to treat lung cancer (nct00809237) (6) Other clinical trials are available at clinicaltrials.gov
Simvastatin	Antihyperlipidemic	(1) Simvastatin plus dual anti-HER2 therapy for metastatic breast cancer (nct03324425) (2) Trial of xp (capecitabine/cddp) simvastatin in advanced gastric cancer patients (nct0109908) (3) Simvastatin in preventing a new breast cancer in women at high risk for a new breast cancer (nct00334542) (4) Metformin and simvastatin use in bladder cancer (nct02360618) (5) A phase I study of high-dose simvastatin in patients with gastrointestinal tract cancer who failed to standard chemotherapy (nct03086291) (6) Other clinical trials are available at clinicaltrials.gov
Digoxin	Antiheart failure	(1) Potentiation of cisplatin-based chemotherapy by digoxin in advanced unresectable head and neck cancer patients (nct02906800) (2) Digoxin for recurrent prostate cancer (nct01162135) (3) Capecitabine with digoxin for metastatic breast cancer (nct01887288) (4) Phase IB metformin, digoxin, and simvastatin in solid tumors (nct03889795) (5) Phase II multicenter study of digoxin per os in classic or endemic Kaposi's sarcoma (nct02212639) (6) Other clinical trials are available at clinicaltrials.gov
Fluphenazine	Antipsychotics	(1) Fluphenazine in treating patients with refractory advanced multiple myeloma (nct00335647) (2) Study of fluphenazine in relapsed or relapsed-and-refractory multiple myeloma (nct00821301)

## References

[1] Siegel R. L., Miller K. D., Fuchs H. E., Jemal A. (2021). Cancer statistics, 2021. *CA: a Cancer Journal for Clinicians*.

[2] Koren E., Fuchs Y. (2021). Modes of regulated cell death in cancer. *Cancer Discovery*.

[3] Gonzalez-Fierro A., Duenas-Gonzalez A. (2021). Drug repurposing for cancer therapy, easier said than done. *Seminars in Cancer Biology*.

[4] Bray F., Ferlay J., Soerjomataram I. (2018). GLOBOCAN estimates of incidence and mortality worldwide for 36 cancers in 185 countries. *CA: a Cancer Journal for Clinicians*.

[5] Brown A. S., Patel C. J. (2017). A standard database for drug repositioning. *Scientific data*.

[6] Wood S. L., Pernemalm M., Crosbie P. A., Whetton A. D. (2015). Molecular histology of lung cancer: from targets to treatments. *Cancer Treatment Reviews*.

[7] Hill A., Gotham D., Fortunak J. (2016). Target prices for mass production of tyrosine kinase inhibitors for global cancer treatment. *BMJ Open*.

[8] Bedard P. L., Hyman D. M., Davids M. S., Siu L. L. (2020). Small molecules, big impact: 20 years of targeted therapy in oncology. *Lancet*.

[9] Pammolli F., Magazzini L., Riccaboni M. (2011). The productivity crisis in pharmaceutical R&D. *Nature Reviews. Drug Discovery*.

[10] Waring M. J., Arrowsmith J., Leach A. R. (2015). An analysis of the attrition of drug candidates from four major pharmaceutical companies. *Nature Reviews. Drug Discovery*.

[11] Hanash S., Taguchi A. (2010). The grand challenge to decipher the cancer proteome. *Nature Reviews Cancer*.

[12] Nosengo N. (2016). Can you teach old drugs new tricks?. *Nature*.

[13] Hay M., Thomas D. W., Craighead J. L., Economides C., Rosenthal J. (2014). Clinical development success rates for investigational drugs. *Nature Biotechnology*.

[14] Ashburn T. T., Thor K. B. (2004). Drug repositioning: identifying and developing new uses for existing drugs. *Nature Reviews Drug Discovery*.

[15] Pushpakom S., Iorio F., Eyers P. A. (2019). Drug repurposing: progress, challenges and recommendations. *Nature Reviews. Drug Discovery*.

[16] Kirtonia A., Gala K., Fernandes S. G. (2021). Repurposing of drugs: an attractive pharmacological strategy for cancer therapeutics. *Seminars in Cancer Biology*.

[17] Singhal S., Mehta J., Desikan R. (1999). Antitumor activity of thalidomide in refractory multiple myeloma. *The New England Journal of Medicine*.

[18] Sivasankaran T. G., Udayakumar R., Elanchezhiyan C., Sabhanayakam S. (2008). Effect of sildenafil citrate (Viagra) and ethanol on the albino rat testis: a scanning electron microscopic approach. *Cell Biology International*.

[19] Turanli B., Altay O., Boren J. (2019). Systems biology based drug repositioning for development of cancer therapy. *Seminars in cancer biology*.

[20] Hayes J. D., Dinkova-Kostova A. T., Tew K. D. (2020). Oxidative stress in cancer. *Cancer Cell*.

[21] Gorrini C., Harris I. S., Mak T. W. (2013). Modulation of oxidative stress as an anticancer strategy. *Nature Reviews. Drug Discovery*.

[22] Dharmaraja A. T. (2017). Role of reactive oxygen species (ROS) in therapeutics and drug resistance in cancer and bacteria. *Journal of Medicinal Chemistry*.

[23] Sousa J. S., D'Imprima E., Vonck J. (2018). Mitochondrial respiratory chain complexes. *Subcellular Biochemistry*.

[24] Wang Y., Qi H., Liu Y. (2021). The double-edged roles of ROS in cancer prevention and therapy. *Theranostics*.

[25] Fridovich I. (1983). Superoxide radical: an endogenous toxicant. *Annual Review of Pharmacology and Toxicology*.

[26] Imlay J. A. (2013). The molecular mechanisms and physiological consequences of oxidative stress: lessons from a model bacterium. *Nature Reviews. Microbiology*.

[27] Del Rio L. A., Lopez-Huertas E. (2016). ROS generation in peroxisomes and its role in cell signaling. *Plant & Cell Physiology*.

[28] Yoboue E. D., Sitia R., Simmen T. (2018). Redox crosstalk at endoplasmic reticulum (ER) membrane contact sites (MCS) uses toxic waste to deliver messages. *Cell Death & Disease*.

[29] Sies H., Jones D. P. (2020). Reactive oxygen species (ROS) as pleiotropic physiological signalling agents. *Nature Reviews. Molecular Cell Biology*.

[30] Winterbourn C. C., Kettle A. J., Hampton M. B. (2016). Reactive oxygen species and neutrophil function. *Annual Review of Biochemistry*.

[31] Dickinson B. C., Chang C. J. (2011). Chemistry and biology of reactive oxygen species in signaling or stress responses. *Nature Chemical Biology*.

[32] Urso L., Cavallari I., Sharova E., Ciccarese F., Pasello G., Ciminale V. (2020). Metabolic rewiring and redox alterations in malignant pleural mesothelioma. *British Journal of Cancer*.

[33] de Jager T. L., Cockrell A. E., Du Plessis S. S. (2017). Ultraviolet light induced generation of reactive oxygen species. *Advances in Experimental Medicine and Biology*.

[34] Kumari S., Badana A. K., Malla R. (2018). Reactive oxygen species: a key constituent in cancer survival. *Biomarker Insights*.

[35] Inoue M., Sato E. F., Nishikawa M. (2003). Mitochondrial generation of reactive oxygen species and its role in aerobic life. *Current Medicinal Chemistry*.

[36] Rhee S. G. (1999). Redox signaling: hydrogen peroxide as intracellular messenger. *Experimental & Molecular Medicine*.

[37] Stone J. R., Yang S. (2006). Hydrogen peroxide: a signaling messenger. *Antioxidants & Redox Signaling*.

[38] Go Y. M., Chandler J. D., Jones D. P. (2015). The cysteine proteome. *Free Radical Biology & Medicine*.

[39] Ursini F., Maiorino M., Forman H. J. (2016). Redox homeostasis: the golden mean of healthy living. *Redox Biology*.

[40] Niki E. (2016). Oxidative stress and antioxidants: *Distress* or *eustress*?. *Archives of Biochemistry and Biophysics*.

[41] Sies H. (2017). Hydrogen peroxide as a central redox signaling molecule in physiological oxidative stress: oxidative eustress. *Redox Biology*.

[42] Chance B., Sies H., Boveris A. (1979). Hydroperoxide metabolism in mammalian organs. *Physiological Reviews*.

[43] Zeida A., Trujillo M., Ferrer-Sueta G., Denicola A., Estrin D. A., Radi R. (2019). Catalysis of peroxide reduction by fast reacting protein thiols. *Chemical Reviews*.

[44] Poole L. B. (2015). The basics of thiols and cysteines in redox biology and chemistry. *Free Radical Biology & Medicine*.

[45] Naik E., Dixit V. M. (2011). Mitochondrial reactive oxygen species drive proinflammatory cytokine production. *The Journal of Experimental Medicine*.

[46] Gloire G., Legrand-Poels S., Piette J. (2006). NF-*κ*B activation by reactive oxygen species: Fifteen years later. *Biochemical Pharmacology*.

[47] Halliwell B. (2007). Biochemistry of oxidative stress. *Biochemical Society Transactions*.

[48] Cui Q., Wang J. Q., Assaraf Y. G. (2018). Modulating ROS to overcome multidrug resistance in cancer. *Drug Resistance Updates*.

[49] Stadtman E. R., Levine R. L. (2003). Free radical-mediated oxidation of free amino acids and amino acid residues in proteins. *Amino Acids*.

[50] Ishikawa K., Takenaga K., Akimoto M. (2008). ROS-generating mitochondrial DNA mutations can regulate tumor cell metastasis. *Science*.

[51] Brovkovych V., Aldrich A., Li N., Atilla-Gokcumen G. E., Frasor J. (2019). Removal of serum lipids and lipid-derived metabolites to investigate breast cancer cell biology. *Proteomics*.

[52] Gaschler M. M., Stockwell B. R. (2017). Lipid peroxidation in cell death. *Biochemical and Biophysical Research Communications*.

[53] Ayala A., Munoz M. F., Arguelles S. (2014). Lipid peroxidation: production, metabolism, and signaling mechanisms of malondialdehyde and 4-hydroxy-2-nonenal. *Oxidative Medicine and Cellular Longevity*.

[54] Maiorino M., Conrad M., Ursini F. (2018). GPx4, lipid peroxidation, and cell death: discoveries, rediscoveries, and open issues. *Antioxidants & Redox Signaling*.

[55] Gamcsik M. P., Kasibhatla M. S., Teeter S. D., Colvin O. M. (2012). Glutathione levels in human tumors. *Biomarkers*.

[56] Harris I. S., DeNicola G. M. (2020). The complex interplay between antioxidants and ROS in cancer. *Trends in Cell Biology*.

[57] Aruoma O. I., Grootveld M., Bahorun T. (2006). Free radicals in biology and medicine: from inflammation to biotechnology. *BioFactors*.

[58] Nguyen N. H., Tran G. B., Nguyen C. T. (2020). Anti-oxidative effects of superoxide dismutase 3 on inflammatory diseases. *Journal of Molecular Medicine*.

[59] Kirkman H. N., Gaetani G. F. (2007). Mammalian catalase: a venerable enzyme with new mysteries. *Trends in Biochemical Sciences*.

[60] Couto N., Wood J., Barber J. (2016). The role of glutathione reductase and related enzymes on cellular redox homoeostasis network. *Free Radical Biology & Medicine*.

[61] Bian M., Fan R., Zhao S., Liu W. (2019). Targeting the thioredoxin system as a strategy for cancer therapy. *Journal of Medicinal Chemistry*.

[62] Zhang J., Li X., Han X., Liu R., Fang J. (2017). Targeting the thioredoxin system for cancer therapy. *Trends in Pharmacological Sciences*.

[63] Harris I. S., Treloar A. E., Inoue S. (2015). Glutathione and thioredoxin antioxidant pathways synergize to drive cancer initiation and progression. *Cancer Cell*.

[64] Marinho H. S., Real C., Cyrne L., Soares H., Antunes F. (2014). Hydrogen peroxide sensing, signaling and regulation of transcription factors. *Redox Biology*.

[65] Hayes J. D., Dinkova-Kostova A. T. (2014). The Nrf2 regulatory network provides an interface between redox and intermediary metabolism. *Trends in Biochemical Sciences*.

[66] Sies H., Berndt C., Jones D. P. (2017). Oxidative Stress. *Annual Review of Biochemistry*.

[67] Deshmukh P., Unni S., Krishnappa G., Padmanabhan B. (2017). The Keap1-Nrf2 pathway: promising therapeutic target to counteract ROS-mediated damage in cancers and neurodegenerative diseases. *Biophysical Reviews*.

[68] Sánchez-Álvarez M., Strippoli R., Donadelli M., Bazhin A. V., Cordani M. (2019). Sestrins as a therapeutic bridge between ROS and autophagy in cancer. *Cancers*.

[69] Rhee S. G., Bae S. H. (2015). The antioxidant function of sestrins is mediated by promotion of autophagic degradation of Keap1 and Nrf2 activation and by inhibition of mTORC1. *Free Radical Biology & Medicine*.

[70] Eijkelenboom A., Burgering B. M. (2013). FOXOs: signalling integrators for homeostasis maintenance. *Nature Reviews. Molecular Cell Biology*.

[71] Klotz L. O., Sanchez-Ramos C., Prieto-Arroyo I., Urbanek P., Steinbrenner H., Monsalve M. (2015). Redox regulation of FoxO transcription factors. *Redox Biology*.

[72] Hernansanz-Agustin P., Ramos E., Navarro E. (2017). Mitochondrial complex I deactivation is related to superoxide production in acute hypoxia. *Redox Biology*.

[73] Kaelin W. G., Ratcliffe P. J. (2008). Oxygen sensing by metazoans: the central role of the HIF hydroxylase pathway. *Molecular Cell*.

[74] Oliveira-Marques V., Marinho H. S., Cyrne L., Antunes F. (2009). Role of hydrogen peroxide in NF-kappaB activation: from inducer to modulator. *Antioxidants & Redox Signaling*.

[75] Schreck R., Rieber P., Baeuerle P. A. (1991). Reactive oxygen intermediates as apparently widely used messengers in the activation of the NF-kappa B transcription factor and HIV-1. *The EMBO Journal*.

[76] Halvey P. J., Hansen J. M., Johnson J. M., Go Y. M., Samali A., Jones D. P. (2007). Selective oxidative stress in cell nuclei by nuclear-targeted D-amino acid oxidase. *Antioxidants & Redox Signaling*.

[77] Maillet A., Pervaiz S. (2012). Redox regulation of p53, redox effectors regulated by p53: a subtle balance. *Antioxidants & Redox Signaling*.

[78] Nguyen T. T., Grimm S. A., Bushel P. R. (2018). Revealing a human p53 universe. *Nucleic Acids Research*.

[79] Liu B., Chen Y., St. Clair D. K. (2008). ROS and p53: a versatile partnership. *Free Radical Biology & Medicine*.

[80] Herzig S., Shaw R. J. (2018). AMPK: guardian of metabolism and mitochondrial homeostasis. *Nature Reviews. Molecular Cell Biology*.

[81] Soriano F. X., Baxter P., Murray L. M., Sporn M. B., Gillingwater T. H., Hardingham G. E. (2009). Transcriptional regulation of the AP-1 and Nrf2 target gene sulfiredoxin. *Molecules and Cells*.

[82] Kovacs D., Sigmond T., Hotzi B. (2019). HSF1Base: a comprehensive database of HSF1 (heat shock factor 1) target genes. *International Journal of Molecular Sciences*.

[83] St-Pierre J., Drori S., Uldry M. (2006). Suppression of reactive oxygen species and neurodegeneration by the PGC-1 transcriptional coactivators. *Cell*.

[84] Echtay K. S., Roussel D., St-Pierre J. (2002). Superoxide activates mitochondrial uncoupling proteins. *Nature*.

[85] Londhe A. D., Bergeron A., Curley S. M. (2020). Regulation of PTP1B activation through disruption of redox-complex formation. *Nature Chemical Biology*.

[86] An H., Heo J. S., Kim P. (2021). Tetraarsenic hexoxide enhances generation of mitochondrial ROS to promote pyroptosis by inducing the activation of caspase-3/GSDME in triple-negative breast cancer cells. *Cell Death & Disease*.

[87] Kirtonia A., Sethi G., Garg M. (2020). The multifaceted role of reactive oxygen species in tumorigenesis. *Cellular and Molecular Life Sciences*.

[88] Kirkpatrick D. L., Powis G. (2017). Clinically evaluated cancer drugs inhibiting redox signaling. *Antioxidants & Redox Signaling*.

[89] Ralph S. J., Nozuhur S., ALHulais R. A., Rodríguez-Enríquez S., Moreno-Sánchez R. (2019). Repurposing drugs as pro-oxidant redox modifiers to eliminate cancer stem cells and improve the treatment of advanced stage cancers. *Medicinal Research Reviews*.

[90] Sassetti E., Clausen M. H., Laraia L. (2021). Small-molecule inhibitors of reactive oxygen species production. *Journal of Medicinal Chemistry*.

[91] Bridge G., Rashid S., Martin S. (2014). DNA mismatch repair and oxidative DNA damage: implications for cancer biology and treatment. *Cancers*.

[92] Pauling L. (1980). Vitamin C therapy of advanced cancer. *The New England Journal of Medicine*.

[93] Chen Q., Espey M. G., Sun A. Y. (2007). Ascorbate in pharmacologic concentrations selectively generates ascorbate radical and hydrogen peroxide in extracellular fluid in vivo. *Proceedings of the National Academy of Sciences of the United States of America*.

[94] Wright M. E., Mayne S. T., Stolzenberg-Solomon R. Z. (2004). Development of a comprehensive dietary antioxidant index and application to lung cancer risk in a cohort of male smokers. *American Journal of Epidemiology*.

[95] Du J., Martin S. M., Levine M. (2010). Mechanisms of ascorbate-induced cytotoxicity in pancreatic cancer. *Clinical Cancer Research*.

[96] Espey M. G., Chen P., Chalmers B. (2011). Pharmacologic ascorbate synergizes with gemcitabine in preclinical models of pancreatic cancer. *Free Radical Biology & Medicine*.

[97] Stephenson C. M., Levin R. D., Spector T., Lis C. G. (2013). Phase I clinical trial to evaluate the safety, tolerability, and pharmacokinetics of high-dose intravenous ascorbic acid in patients with advanced cancer. *Cancer Chemotherapy and Pharmacology*.

[98] Banhegyi G., Benedetti A., Margittai E. (2014). Subcellular compartmentation of ascorbate and its variation in disease states. *Biochimica et Biophysica Acta*.

[99] Naidu K. A. (2003). Vitamin C in human health and disease is still a mystery? An overview. *Nutrition Journal*.

[100] Zasowska-Nowak A., Nowak P. J., Cialkowska-Rysz A. (2021). High-dose vitamin C in advanced-stage cancer patients. *Nutrients*.

[101] Kazmierczak-Baranska J., Boguszewska K., Adamus-Grabicka A., Karwowski B. T. (2020). Two faces of vitamin C-antioxidative and pro-oxidative agent. *Nutrients*.

[102] Zingg J. M. (2019). Vitamin E: regulatory role on signal transduction. *IUBMB Life*.

[103] Azzi A. (2018). Many tocopherols, one vitamin E. *Molecular Aspects of Medicine*.

[104] Ricciarelli R., Azzi A. (1998). Regulation of Recombinant PKC*α* Activity by Protein Phosphatase 1 and Protein Phosphatase 2A. *Archives of Biochemistry and Biophysics*.

[105] Nogueira V., Park Y., Chen C. C. (2008). Akt determines replicative senescence and oxidative or oncogenic premature senescence and sensitizes cells to oxidative apoptosis. *Cancer Cell*.

[106] Huang P. H., Chuang H. C., Chou C. C. (2013). Vitamin E facilitates the inactivation of the kinase Akt by the phosphatase PHLPP1. *Science Signaling*.

[107] Ungurianu A., Zanfirescu A., Nițulescu G., Margină D. (2021). Vitamin E beyond its antioxidant label. *Antioxidants*.

[108] Cardenas E., Ghosh R. (2013). Vitamin E: a dark horse at the crossroad of cancer management. *Biochemical Pharmacology*.

[109] Wiel C., Le Gal K., Ibrahim M. X. (2019). BACH1 stabilization by antioxidants stimulates lung cancer metastasis. *Cell*.

[110] Tasina E., Haidich A. B., Kokkali S., Arvanitidou M. (2011). Efficacy and safety of tigecycline for the treatment of infectious diseases: a meta-analysis. *The Lancet Infectious Diseases*.

[111] Stein G. E., Babinchak T. (2013). Tigecycline: an update. *Diagnostic Microbiology and Infectious Disease*.

[112] Cai Y., Bai N., Liu X., Liang B., Wang J., Wang R. (2016). Tigecycline: alone or in combination?. *Infectious Diseases*.

[113] Jia X., Gu Z., Chen W., Jiao J. (2016). Tigecycline targets nonsmall cell lung cancer through inhibition of mitochondrial function. *Fundamental & Clinical Pharmacology*.

[114] Skrtic M., Sriskanthadevan S., Jhas B. (2011). Inhibition of mitochondrial translation as a therapeutic strategy for human acute myeloid leukemia. *Cancer Cell*.

[115] Tan J., Song M., Zhou M., Hu Y. (2017). Antibiotic tigecycline enhances cisplatin activity against human hepatocellular carcinoma through inducing mitochondrial dysfunction and oxidative damage. *Biochemical and Biophysical Research Communications*.

[116] Bahuguna A., Rawat D. S. (2020). An overview of new antitubercular drugs, drug candidates, and their targets. *Medicinal Research Reviews*.

[117] Song M., Wu H., Wu S. (2016). Antibiotic drug levofloxacin inhibits proliferation and induces apoptosis of lung cancer cells through inducing mitochondrial dysfunction and oxidative damage. *Biomedicine & Pharmacotherapy*.

[118] Yu M., Li R., Zhang J. (2016). Repositioning of antibiotic levofloxacin as a mitochondrial biogenesis inhibitor to target breast cancer. *Biochemical and Biophysical Research Communications*.

[119] Henehan M., Montuno M., De Benedetto A. (2017). Doxycycline as an anti-inflammatory agent: updates in dermatology. *Journal of the European Academy of Dermatology and Venereology*.

[120] Markowska A., Kaysiewicz J., Markowska J., Huczynski A. (2019). Doxycycline, salinomycin, monensin and ivermectin repositioned as cancer drugs. *Bioorganic & Medicinal Chemistry Letters*.

[121] Tan Q., Yan X., Song L. (2017). Induction of mitochondrial dysfunction and oxidative damage by antibiotic drug doxycycline enhances the responsiveness of glioblastoma to chemotherapy. *Medical Science Monitor*.

[122] Shieh J. M., Huang T. F., Hung C. F., Chou K. H., Tsai Y. J., Wu W. B. (2010). Activation of c-Jun N-terminal kinase is essential for mitochondrial membrane potential change and apoptosis induced by doxycycline in melanoma cells. *British Journal of Pharmacology*.

[123] Tong F., Ye Y., Chen B. (2020). Bone-targeting prodrug mesoporous silica-based nanoreactor with reactive oxygen species burst for enhanced chemotherapy. *ACS Applied Materials & Interfaces*.

[124] Mark T. M., Coleman M. (2016). It's time to take clarithromycin seriously in multiple myeloma. *Acta Haematologica*.

[125] Zhou B., Xia M., Wang B. (2019). Clarithromycin synergizes with cisplatin to inhibit ovarian cancer growth in vitro and in vivo. *Journal of Ovarian Research*.

[126] Xu J., Shi P. Y., Li H., Zhou J. (2020). Broad spectrum antiviral agent niclosamide and its therapeutic potential. *ACS Infectious Diseases*.

[127] Jin Y., Lu Z., Ding K. (2010). Antineoplastic mechanisms of niclosamide in acute myelogenous leukemia stem cells: inactivation of the NF-kappaB pathway and generation of reactive oxygen species. *Cancer Research*.

[128] Zhao J., He Q., Gong Z., Chen S., Cui L. (2016). Niclosamide suppresses renal cell carcinoma by inhibiting Wnt/*β*-catenin and inducing mitochondrial dysfunctions. *Springerplus*.

[129] Chen L., Wang L., Shen H., Lin H., Li D. (2017). Anthelminthic drug niclosamide sensitizes the responsiveness of cervical cancer cells to paclitaxel via oxidative stress-mediated mTOR inhibition. *Biochemical and Biophysical Research Communications*.

[130] Lee S. L., Son A. R., Ahn J., Song J. Y. (2014). Niclosamide enhances ROS-mediated cell death through c-Jun activation. *Biomedicine & Pharmacotherapy*.

[131] Lu L., Dong J., Wang L. (2018). Activation of STAT3 and Bcl-2 and reduction of reactive oxygen species (ROS) promote radioresistance in breast cancer and overcome of radioresistance with niclosamide. *Oncogene*.

[132] Akgun O., Erkisa M., Ari F. (2019). Effective and new potent drug combination: histone deacetylase and Wnt/*β*‐catenin pathway inhibitors in lung carcinoma cells. *Journal of Cellular Biochemistry*.

[133] Nath J., Paul R., Ghosh S. K., Paul J., Singha B., Debnath N. (2020). Drug repurposing and relabeling for cancer therapy: emerging benzimidazole antihelminthics with potent anticancer effects. *Life Sciences*.

[134] Castro L. S., Kviecinski M. R., Ourique F. (2016). Albendazole as a promising molecule for tumor control. *Redox Biology*.

[135] Wang L. J., Lee Y. C., Huang C. H. (2019). Non-mitotic effect of albendazole triggers apoptosis of human leukemia cells via SIRT3/ROS/p38 MAPK/TTP axis-mediated TNF-*α* upregulation. *Biochemical Pharmacology*.

[136] Patel O. P. S., Beteck R. M., Legoabe L. J. (2021). Exploration of artemisinin derivatives and synthetic peroxides in antimalarial drug discovery research. *European Journal of Medicinal Chemistry*.

[137] Cheong D. H. J., Tan D. W. S., Wong F. W. S., Tran T. (2020). Anti-malarial drug, artemisinin and its derivatives for the treatment of respiratory diseases. *Pharmacological Research*.

[138] Xiao F. L., Gao W. J., Liu C. Y., Wang X. P., Chen T. S. (2011). Artemisinin induces caspase-8/9-mediated and Bax/Bak-independent apoptosis in human lung adenocarcinoma (ASTC-a-1) cells. *Journal of X-Ray Science and Technology*.

[139] Li P., Yang S., Dou M., Chen Y., Zhang J., Zhao X. (2014). Synergic effects of artemisinin and resveratrol in cancer cells. *Journal of Cancer Research and Clinical Oncology*.

[140] Noori S., Hassan Z. M., Farsam V. (2014). Artemisinin as a Chinese medicine, selectively induces apoptosis in pancreatic tumor cell line. *Chinese Journal of Integrative Medicine*.

[141] Fox J. M., Moynihan J. R., Mott B. T. (2016). Artemisinin-derived dimer ART-838 potently inhibited human acute leukemias, persisted in vivo, and synergized with antileukemic drugs. *Oncotarget*.

[142] Jia J., Qin Y., Zhang L. (2016). Artemisinin inhibits gallbladder cancer cell lines through triggering cell cycle arrest and apoptosis. *Molecular Medicine Reports*.

[143] Gomez V. E., Giovannetti E., Peters G. J. (2015). Unraveling the complexity of autophagy: potential therapeutic applications in pancreatic ductal adenocarcinoma. *Seminars in Cancer Biology*.

[144] Saleem A., Dvorzhinski D., Santanam U. (2012). Effect of dual inhibition of apoptosis and autophagy in prostate cancer. *Prostate*.

[145] Xia H., Green D. R., Zou W. (2021). Autophagy in tumour immunity and therapy. *Nature Reviews. Cancer*.

[146] Gao L., Zhao X., Lang L., Shay C., Andrew Yeudall W., Teng Y. (2018). Autophagy blockade sensitizes human head and neck squamous cell carcinoma towards CYT997 through enhancing excessively high reactive oxygen species-induced apoptosis. *Journal of Molecular Medicine (Berlin, Germany)*.

[147] Vera-Ramirez L., Vodnala S. K., Nini R., Hunter K. W., Green J. E. (2018). Autophagy promotes the survival of dormant breast cancer cells and metastatic tumour recurrence. *Nature Communications*.

[148] Di Bello E., Zwergel C., Mai A., Valente S. (2020). The innovative potential of statins in cancer: new targets for new therapies. *Frontiers in Chemistry*.

[149] Sánchez C. A., Rodríguez E., Varela E. (2008). Statin-induced inhibition of MCF-7 breast cancer cell proliferation is related to cell cycle arrest and apoptotic and necrotic cell death mediated by an enhanced oxidative stress. *Cancer Investigation*.

[150] Castellanos-Esparza Y. C., Wu S., Huang L. (2018). Synergistic promoting effects of pentoxifylline and simvastatin on the apoptosis of triple-negative MDA-MB-231 breast cancer cells. *International Journal of Oncology*.

[151] Buranrat B., Senggunprai L., Prawan A., Kukongviriyapan V. (2016). Simvastatin and atorvastatin as inhibitors of proliferation and inducers of apoptosis in human cholangiocarcinoma cells. *Life Sciences*.

[152] Alevizopoulos K., Calogeropoulou T., Lang F., Stournaras C. (2014). Na+/K+ ATPase inhibitors in cancer. *Current Drug Targets*.

[153] Wang Z., Zheng M., Li Z. (2009). Cardiac glycosides inhibit p53 synthesis by a mechanism relieved by Src or MAPK inhibition. *Cancer Research*.

[154] Wang T., Xu P., Wang F. (2017). Effects of digoxin on cell cycle, apoptosis and NF-*κ*B pathway in Burkitt's lymphoma cells and animal model. *Leukemia & Lymphoma*.

[155] Wei D., Peng J. J., Gao H. (2013). Digoxin downregulates NDRG1 and VEGF through the inhibition of HIF-1*α* under hypoxic conditions in human lung adenocarcinoma A549 cells. *International Journal of Molecular Sciences*.

[156] Gayed B. A., O'Malley K. J., Pilch J., Wang Z. (2012). Digoxin inhibits blood vessel density and HIF-1a expression in castration-resistant C4-2 xenograft prostate tumors. *Clinical and Translational Science*.

[157] Abu-Remaileh M., Khalaileh A., Pikarsky E., Aqeilan R. I. (2018). WWOX controls hepatic HIF1*α* to suppress hepatocyte proliferation and neoplasia. *Cell Death & Disease*.

[158] Choi E. J., Jung B. J., Lee S. H. (2017). A clinical drug library screen identifies clobetasol propionate as an NRF2 inhibitor with potential therapeutic efficacy in KEAP1 mutant lung cancer. *Oncogene*.

[159] Zhou Y., Zhou Y., Yang M. (2019). Digoxin sensitizes gemcitabine-resistant pancreatic cancer cells to gemcitabine via inhibiting Nrf2 signaling pathway. *Redox Biology*.

[160] Hendouei N., Saghafi F., Shadfar F., Hosseinimehr S. J. (2019). Molecular mechanisms of anti-psychotic drugs for improvement of cancer treatment. *European Journal of Pharmacology*.

[161] Otreba M., Kosmider L. (2021). In vitroanticancer activity of fluphenazine, perphenazine and prochlorperazine. A review. *Journal of Applied Toxicology*.

[162] Xu F., Xia Y., Feng Z. (2019). Repositioning antipsychotic fluphenazine hydrochloride for treating triple negative breast cancer with brain metastases and lung metastases. *American Journal of Cancer Research*.

[163] Menilli L., Garcia-Argaez A. N., Dalla Via L., Miolo G. (2019). The neuroleptic drug fluphenazine induces a significant UVA-mediated cytotoxic effect on three human cancer cell lines through apoptosis. *Photochemical & Photobiological Sciences*.

[164] Mothi M., Sampson S. (2013). Pimozide for schizophrenia or related psychoses. *Cochrane Database of Systematic Reviews*.

[165] Taub R. N., Baker M. A. (1979). Treatment of metastatic malignant melanoma with pimozide. *The Lancet*.

[166] Kim U., Kim C. Y., Lee J. M. (2020). Pimozide inhibits the human prostate cancer cells through the generation of reactive oxygen species. *Frontiers in Pharmacology*.

[167] Cai N., Zhou W., Ye L. L. (2017). The STAT3 inhibitor pimozide impedes cell proliferation and induces ROS generation in human osteosarcoma by suppressing catalase expression. *American Journal of Translational Research*.

[168] Chen J. J., Zhang L. N., Cai N., Zhang Z., Ji K. (2019). Antipsychotic agent pimozide promotes reversible proliferative suppression by inducing cellular quiescence in liver cancer. *Oncology Reports*.

[169] Moreno-Sanchez R., Saavedra E., Gallardo-Perez J. C., Rumjanek F. D., Rodriguez-Enriquez S. (2016). Understanding the cancer cell phenotype beyond the limitations of current omics analyses. *The FEBS Journal*.

[170] Ralph S. J., Pritchard R., Rodríguez-Enríquez S., Moreno-Sánchez R., Ralph R. (2015). Hitting the bull's-eye in metastatic cancers-NSAIDs elevate ROS in mitochondria, inducing malignant cell death. *Pharmaceuticals*.

[171] Kodama R., Kato M., Furuta S. (2013). ROS-generating oxidases Nox1 and Nox4 contribute to oncogenic Ras-induced premature senescence. *Genes to Cells*.

[172] Wang P., Gong Q., Hu J., Li X., Zhang X. (2021). Reactive oxygen species (ROS)-responsive prodrugs, probes, and theranostic prodrugs: applications in the ROS-related diseases. *Journal of Medicinal Chemistry*.

[173] Li Y., Yang J., Sun X. (2021). Reactive oxygen species-based nanomaterials for cancer therapy. *Frontiers in Chemistry*.

[174] Dilruba S., Kalayda G. V. (2016). Platinum-based drugs: past, present and future. *Cancer Chemotherapy and Pharmacology*.

[175] Wheate N. J., Walker S., Craig G. E., Oun R. (2010). The status of platinum anticancer drugs in the clinic and in clinical trials. *Dalton Transactions*.

